# Detection of dengue, west Nile virus, rickettsiosis and leptospirosis by a new real-time PCR strategy

**DOI:** 10.1186/s40064-016-2318-y

**Published:** 2016-05-20

**Authors:** Daniel García-Ruíz, Marco A. Martínez-Guzmán, Albertina Cárdenas-Vargas, Erika Marino-Marmolejo, Abel Gutiérrez-Ortega, Esteban González-Díaz, Rayo Morfin-Otero, Eduardo Rodríguez-Noriega, Hector Pérez-Gómez, Darwin Elizondo-Quiroga

**Affiliations:** Dirección de Biotecnología Médica y Farmacéutica, Centro de Investigación y Asistencia en Tecnología y Diseño del Estado de Jalisco (CIATEJ), Avenida Normalistas No. 800, Colinas de la Normal, C.P. 44270 Guadalajara, Jalisco Mexico; Departamento de Infectología, Hospital Civil Fray Antonio Alcalde, Calle Hospital No. 270, El Retiro, 44200 Guadalajara, Jalisco Mexico

**Keywords:** Dengue, West Nile Virus, Rickettsia, Leptospira, Real time PCR

## Abstract

Dengue virus (DENV) infection causes sudden fever along with several nonspecific signs and symptoms and in severe cases, death. DENV is transmitted to people by *Aedes aegypti and Ae. albopictus* mosquitoes, whose populations increase during rainy season. West Nile Virus (WNV), *Rickettsia* spp. and *Leptospira* spp. are fever-causing pathogens that share many of the initial symptoms of DENV infection and also thrive in the rainy season. Outbreaks in some regions may be due to any of these pathogens that can co-circulate. Plus, they are clinically indistinguishable until severe symptoms appear, even though these diseases should be treated differently. An effective differential diagnosis would help clinicians and vector control departments to make right decisions for control and treatment of these diseases. Therefore, we developed four different SYBR green^**®**^-based reverse transcription quantitative PCR (RT-qPCR) assays for simultaneous detection of DENV, WNV, *Rickettsia* spp. and *Leptospira* spp. The assay has been optimized to yield results in less than 1 h; and in order to reduce contamination risk, all reagents were premixed and lyophilized on 96 well plates and thus only requires the addition of water and total nucleic acids from the sample. Sensitivities of the assays were less than 100 copies of nucleic acid targeted for these four pathogens. Assays did not show cross reactivity with any of the four pathogens nor to human nucleic acids. We are presenting a sensitive and selective kit that detects four relevant pathogens from tropical regions, that is quick, cost-effective and easy to use.

## Background

Dengue fever, West Nile fever, rickettsiosis and leptospirosis are re-emerging infectious diseases worldwide and year-by-year cases are reported in Latin America and other regions, where efforts for epidemiologic surveillance in some cases have limited success. These pathogens produce similar symptoms at the onset of the disease, including: sudden high fever, myalgia, and retro-orbital pain. Despite the presentation of similar symptoms, these diseases progress to different severe outcomes (Bäck and Lundkvist 2013; Levett 2001; Levett et al. 2000; Mahajan 2012; Petersen and Marfin 2002; Sahni and Rydkina 2009). Considering this and also that these agents can share ecological niches, there is potential risk for misdiagnosis and hence silent circulation of some of these pathogens in the aforementioned regions.

The World Health Organization (WHO) classifies dengue disease as classic dengue fever and severe dengue fever. Classic dengue fever includes the symptoms described previously. Severe dengue fever is further divided according to severity into Dengue Hemorrhagic Fever (DHF) and Dengue Shock Syndrome (DSS) (World Health Organization 2009). Infections with WNV in some cases can be fatal; its fever can evolve into a neuroinvasive disease in which paralysis, meningoencephalitis and poliomyelitis-like syndrome may occur (Colpitts et al. 2012). *Rickettsia* spp. replicate inside vascular endothelial cells, causing vascular damage and they can also reach the endothelium of liver, kidneys, pancreas and other organs resulting in internal bleeding (Mahajan 2012). *Leptospira* spp. are spiroquetes that can rapidly move through the blood, reaching the kidneys, heart, lungs and liver. These bacteria damage tissues while moving within organs and can cause multiorganic failure in a few weeks (Levett 2001).

The potentially lethal outcomes of these diseases underline the need for an early differential diagnostic test. Rather than serological assays, molecular technics are required for accurate and sensitive diagnosis, but these technics are time-consuming, expensive, require highly-trained personnel and are not readily available (Cota et al. 2012). Furthermore, an important drawback of molecular technics is the low stability of reagents, particularly enzymes. Lyophilization of reagents has proved to enhance the stability of enzymes at room temperature thus eliminating any special requirements for storage (Lins et al. 2004). Some previously reported protocols for PCR have been designed where all reagents, except the sample, have been lyophilized and tested in-field (Howson et al. 2015; Kamau et al. 2014; Lee et al. 2011). Cryoprotectants like saccharides can be added to enhance the stability. For instance, trehalose (a glucose disaccharide) has been tested and found to have properties that aids to maintain the native structure and function of several proteins (Ohtake and Wang 2011).

Although several molecular assays have been developed for diagnosis of each disease herein described (Denison et al. 2014; Lai et al. 2007; Lee et al. 2011; Li et al. 2011; Papin et al. 2004), none of them have been designed to diagnose these four pathogens simultaneously. The aim of this study was to develop a safe, reproducible, stable, quick and easy-to-use simultaneous differential diagnostic test, which would be able to detect four fever-causing agents that could co-circulate in the same geographic area, using primers for each disease and SYBR Green as a fluorescent dye in order to make it cost-effective.

## Methods

### Design of primers

Primers for WNV, *Rickettsia* spp. and *Leptospira* spp. were designed using *CLC Main Workbench 5* with alignments of highly conserved regions selected for each agent as target sequences. For the alignment of WNV we used eight partial sequences of the envelope protein. For *Leptospira* spp., we aligned 97 sequences of the 16S ribosomal RNA gene from seven species and for *Rickettsia* spp., 24 sequences of *gltA* gene from 11 species were aligned. For DENV we used primers reported by Lai et al. capable of detecting a conserved region across the four serotypes (Lai et al. 2007). All primers were synthesized by IDT**™** (Coralville USA). Primers are shown in Table 1.

**Table 1:** Characteristics and concentration of primers used in this work

Primer name	Primer sequence	Length (pb)	Tm (°C)	ΔG of homodimers at 3′ (Kcal/mol)	ΔG of heterodimers at 3′ (Kcal/mol)	Conc. (nM)
DENV-For	TTGAGTAAACYRTGCTGCCTGTAGCTC	27	58.4–62.3	−6.34	−6.34	80
DENV-Rev	GAGACAGCAGGATCTCTGGTCTYTC	25	58.6–59.8	−6.28		130
WNV-For	GGTGGATTTGGTTCTCGAAGGCG	23	60.4	−3.61	−3.61	60
WNV-Rev	AGGGTCAGCACGTTTGTCATTGTG	24	60	−1.95		120
RICK-For	TATGCTTGCGGCTGTCGGTTCTC	23	61.7	−3.14	−3.61	1000
RICK-Rev	TTGCGGTAAGTTCGTAGTCTGCTTCTT	27	59.9	−3.61		1000
LEP-For	AGCAGCCGCGGTAATACGTATGG	23	61.3	−3.14	−3.55	1000
LEP-Rev	TTTAGGGCGTGGATTACTGGGG	22	59	−0.96		1000

*Tm* melting temperature, *Conc.* concentration used in this study, *ΔG* Gibbs energy

### RT-qPCR design

**Fig. 1:**
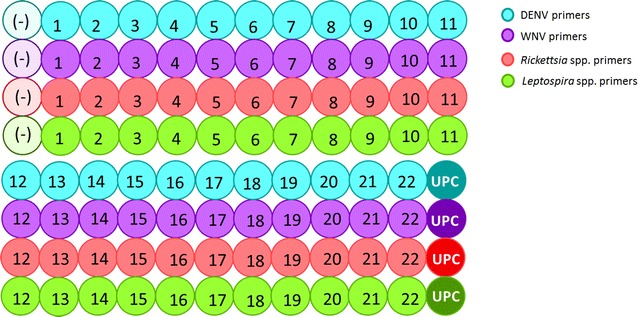
Primer distribution in a 96-well PCR plate. All the wells contain a QuantiFast SYBR Green RT-qPCR Master Mix. Additionally, the wells contain the primers for DENV (*blue*), WNV (*violet*), *Rickettsia* spp. (*red*) and *Leptospira* spp. (*green*). *Numbers* represent the sample that must be added to each set of four wells. The plate includes a set of four wells for negative control [*light-colored wells* with a “(−)” sign] and another for the *Universal Positive Control* (*dark-colored wells* with “UPC”)

RT-qPCRs assays were carried out in a Light Cycler 480 II PCR platform (Roche Diagnostics, Penzberg, Germany), using QuantiFast SYBR Green RT-qPCR Kit (Qiagen™ Hilden Germany). In order to simultaneously detect any of the four pathogens in a sample, a 96-well plate was used with a distribution of each set of primers per agent in a different well of the plate, plus the Master Mix of the QuantiFast kit, and adding 5 µL of total nucleic acids extracted from the sample in each of these four wells; additionally, a no template control was added and positive controls wells were also included (Fig. 1). The concentration of primers were 80 nM forward and 130 nM reverse for DENV, 60 nM forward and 120 nM reverse for WNV, 1000 nM for both primers for Rickettsia and for Leptospira (Table 2). The RT-qPCR conditions were 50 °C for 10 min, then 5 min at 95 °C followed by 40 cycles of 95 °C for 10 s and 60 °C for 30 s; fluorescence quantification was performed during the annealing step (Table 2).

**Table 2:** Conditions for RT-qPCR

Reversal transcription	Cycles:1
Temperature (°C)	Time (min:s)
50	10:00
Activation	Cycles : 1
Temperature (°C)	Time (min:s)
95	5:00
PCR	Cycles : 40
Temperature (°C)	Time (min:s)
95	00:10
60	00:30
Melting curve	Cycles : 1
Temperature (°C)	Time (min:s)
95	00:15
50	00:15
95	NA

*NA* not applies

### Specimens, samples and nucleic acids extraction

For the viral agents positive controls construction, RNA from a serum sample confirmed as DENV-2 by the Health Secretariat of the state of Guerrero, and a WNV horse vaccine donated by the Mexican National Polytechnic Institute, were extracted, using a QiAmp Viral RNA Mini Kit (Qiagen™ Hilden Germany) according to the manufacturer’s instructions. In brief, directly from 140 μL of each specimen, RNAs were extracted and eluted in 60 μL of the elution buffer. *Rickettsia canadensis* (CA410 VR-1444) and *Leptospira interrogans (Stimson) Wenyon (ATCC*^*®*^*23478™*) were purchased from the American Type Culture Collection (ATCC). A 25 cm^2^ tissue flask culture containing fresh Vero cells (ATCC^®^ CCL-81™) was inoculated with *R. canadensis* and maintained at 37 °C with DMEM (GIBCO) medium with 1 % fetal bovine serum. Infected cell cultures were scraped from the flask after 11 days of culture and centrifuged along with the supernatant at 10,000×*g* for 10 min. *L. interrogans* was incubated in 5 mL of EMJH medium at 28 °C for 7 days and then centrifuged at 4000×*g* for 5 min. Pellets for both bacteria were collected and DNA was extracted using QIAmp DNA Mini Kit (Qiagen™ Hilden, Germany) according to the manufacturer’s instructions. In brief, DNAs were extracted and eluted in 200 μL of the elution buffer directly from 200 μL of each specimen.

**Fig. 2:**
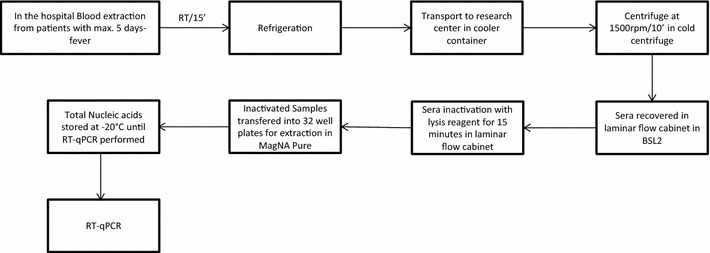
Blood samples were obtained from patients presenting fever during up to 5 days and were left at room temperature (RT) for 15 min for clotting. Samples were then transported from the hospital to the research center in a refrigerated container. Samples were centrifuged at 2000 rpm for 10 min in a cold centrifuge and recovered from the tubes inside a vertical laminar flow cabinet Class II Type A2 in BSL2 facilities. Lysis reagent of MagNA Pure LC total nucleic acid isolation kit was added to each sample and incubated for 15 min inside the laminar flow cabinet. 300 μL of inactivated serum were transferred into 32 well-plates for Roche MagNA Pure LC instrument. Total nucleic acids extractions were stored at −20 °C until RT-qPCR were performed

In order to pre-validate the diagnostic strategy, sera samples from patients showing dengue-like symptoms (less than 5 days since fever onset), were provided by Hospital Civil de *Guadalajara Fray Antonio Alcalde*. The samples were managed in cold conditions at all times and processed in a laminar flow cabinet Class II Type A2 in BSL2 facilities until inactivation. Total nucleic acids extractions from these samples were performed with Roche MagNA Pure LC automated nucleic acid extraction system, using the MagNA Pure LC total nucleic acid isolation kit, according to the manufacturer’s instructions (Roche™ Basel, Switzerland) (Fig. 2). In brief, total nucleic acids were extracted and eluted in 50 μL of elution buffer, directly from 250 μL of each sample.

### Positive controls

For DENV and WNV, reverse transcription reactions were carried out using SuperScript^®^ III Reverse Transcriptase (Invitrogen™ Carlsbad USA). Viral cDNA and bacterial DNA were then amplified by PCR using *TaqPol* (Qiagen™ Hilden Germany) following manufacturer’s instructions. In order to generate positive controls, PCR products were cloned into pCR 2.1 vectors using the TOPO^®^ TA Cloning^®^ Kit (Invitrogen™Carlsbad USA). In brief, 2 μL of PCR product were mixed with 0.5 μL of pCR2.1-TOPO vector, 0.5 μL of NaCl 300 mM, 0.5 μL of MgCl_2_ 15 mM; after incubation for 10 min, electrocompetent *Escherichia coli* TG1 were added and exposed to 1.5 kV electric shock. Plasmidic DNA was extracted using QIAprep Spin Miniprep Kit (Qiagen™ Hilden German) and sequenced by the National Genomics Laboratory for Biodiversity (LANGEBIO, CINVESTAV). The individual controls were mixed together in order to generate a universal positive control with equal Cycle thresholds (C_t_) for each assay. This was achieved by running a gradient from 10^6^ to 10^1^ molecules of target copies for each set of primers in qPCR reaction. Then we calculated the necessary number of each control to amplify in Ct 22.

### Analytical sensitivity and specificity

In order to evaluate the analytical sensitivity, the range of linear detection for each RT-qPCR assay was calculated performing serial dilutions from 10^6^ up to 10^0^ molecules of each positive control per reaction. Additionally, the limit of detection (LOD) was calculated for each RT-qPCR assay by PROBIT analysis. Ten replicas of RT-qPCR amplifications were carried out using solutions with 100, 50, 10 and 1 molecules/μL of each positive control plasmid; 1 μL of each solution was added per replica. The molecular weight of each plasmid with the insert, were used to calculate the number of plasmid molecules in the solutions.

To assess the analytical specificity of the four sets of primers in silico, BLAST analysis was conducted. To discard cross-reactivity, RT-qPCRs assays were performed with primers for WNV, Rickettsia and Leptospira using RNA from DENV2 and *Homo sapiens*, as well as DNA from *Leptospira interrogans*, *Rickettsia canadensis* and *H. sapiens*. The DENV primers were previously tested for cross-reactivity (Lai et al. 2007).

### Lyophilization assays

In order to simplify the process of sample analysis, and diminish the time required for qPCR, we lyophilized all the reagents with rickettsia primers (as an example of primers), using four different cryoprotectors treatments. This experiment was required to test the stability of the reverse transcriptase and polymerase enzymes after the lyophilization process and not for the primers stability, since they are commercially distributed in this presentation. Treatments were: trehalose 0 %, trehalose 2.5 % w/v, trehalose 5 % w/v and trehalose 10 % w/v. The plate containing the reactions was incubated at −80 °C overnight, and then maintained in a lyophilization chamber for 24 h at a pressure lower than 0.133 mBar at −40 C. After lyophilization, a fresh reaction without cryoprotectors was included as a positive control.

### Statistical analysis

Sample size was calculated using a confidence level of 95 and 10 % margin of error for the DENV-positive cases in the metropolitan area of Guadalajara, Jalisco, Mexico, in 2014 (‘Secretaria de Salud del Estado de Jalisco. Prevención Dengue’ 2015). Statistical analysis for qPCR assays were performed as follows: efficiency was calculated in the Roche LightCycler 480 II software, linearity range and regression analysis were determined by Microsoft Excel 2013, and LOD was estimated by Probit analysis using MedCalc software for Windows version 16.4 (MedCalc Software, Ostend, Belgium).

## Results

### Primers

For DENV we used primers reported by Lai et al. (2007); these primers amplify a 258 bp fragment of the 3′ untranslated region. For WNV, *Rickettsia* spp. and *Leptospira* spp, a pair of primers was designed in order to amplify conserved regions of each agent. The WNV primers target a 210 bp region of the gene coding for the envelope protein, Leptospire primers amplify a 297 bp region of *16 rrs* gene and Rickettsia primers, amplify an 83 bp region of the *gltA* gene. These primers are predicted to identify hundreds of strains reported for each pathogen (Table 3).

**Table 3:** Number of retrieved sequences for each specie, genome species or type of virus; in *Rickettsia* spp., symbionts and endosymbionts were not included

Disease	Agent	Number of retrieved sequences
Rickettsiosis	*R. prowazekii*	12
	*R. rickettsii*	15
	*R. typhi*	4
	*Israeli tick typhus rickettsia*	3
	*R. felis*	3
	*R. conorii*	5
	*R. parkeri*	5
	*R. rhipicephali*	3
	*R. akari*	2
	*R. honei*	2
	*R. canadensis*	4
	*R. bellii*	11
	*R. massiliae*	5
	*Candidatus R. amblyommii*	6
	*R. montanensis*	1
	*R. slovaca*	5
	*R. peacockii*	2
	*R. monacensis*	1
	*R. tamurae*	1
	*R. tarasevichiae*	4
	*R. philipii*	1
	*R. africae*	6
	*R. japonica*	2
	*R. heilongjiangensis*	7
	*R. aeschlimannii*	10
	*R. sibirica*	4
	*Candidatus R. andeanae*	2
	*R. mongolotimonae*	1
	*Candidatus R. kulagini*	1
	*R. marmionii*	1
	*R. montana*	1
	*Candidatus R. antechini*	1
	*Candidatus R. gravesii*	1
	*R. raoultii*	29
	*R. australis*	1
	*Candidatus R. rioja*	1
	*Candidatus R.tasmanensis*	2
	*R. asiatica*	5
	*Candidatus R. rara*	1
	*Candidatus R. uilenbergi*	1
	*Candidatus R. davousti*	1
	*Candidatus R. hoogstraalii*	1
	*R. helvetica*	1
Leptospirosis	*L. interrogans*	178
	*L. borgpeterseni*	64
	*L. santarosai*	21
	*L. noguchii*	18
	*L. weilii*	14
	*L. alexanderi*	5
	*L. kirschneri*	29
	*L. inadai*	5
	*L. meyeri*	8
	*L. fainei*	6
	*L. broomii*	4
	*L. biflexa*	16
	*L. wolffii*	2
	*L. idonii*	1
	*L. licerasiae*	10
	*L. kmetyi*	1
	*L. wolbachi*	3
	*Leptospira* sp.	94
Dengue fever	DENV 1	553
	DENV 2	821
	DENV 3	356
	DENV 4	148
West Nile fever	WNV	1000

### Analytical sensitivity and specificity

**Fig. 3:**
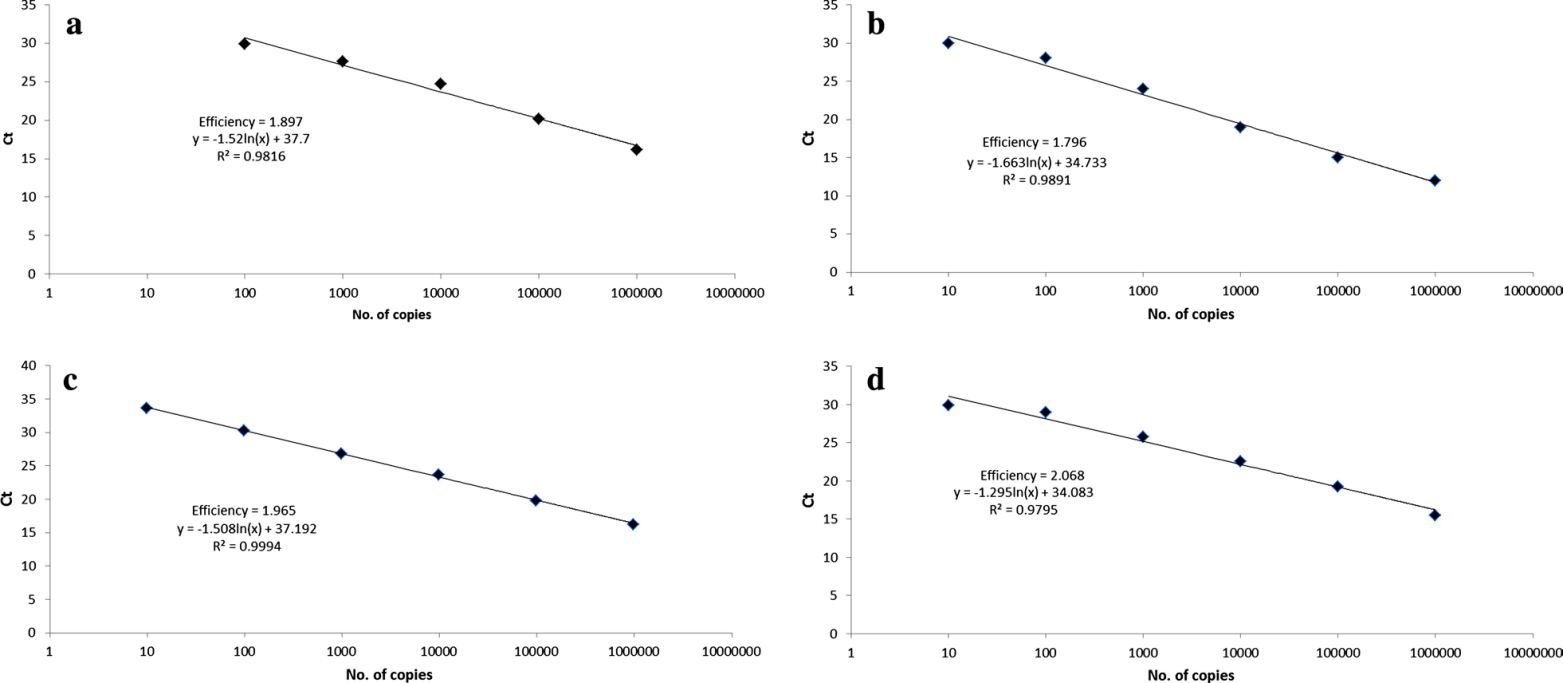
Linearity ranges of the assays. Standard curves of the amplification plots are shown for the assays of Dengue virus (**a**), west Nile virus (**b**), Rickettsia (**c**), and Leptospira (**d**). Serial dilutions of 10^6^–10^0^ molecules of each positive control were used as a template for RT-qPCR reactions. The reactions were carried out in duplicate and Cycle threshold (C_t_), efficiency, and slope were determined according to the *Fix Points* algorithm included in the Lightycler 480 II^®^ software

In order to evaluate the analytical sensitivity of each assay, standard curves were generated by plotting the threshold cycle of each positive control against the concentration measured in copies/µL. Linearity of the assays in this study and their correlation coefficients were as follows: WNV (r^2^ = 0.9891), Rickettsia (r^2^ = 0.9994) and Leptospira (r^2^ = 0.9795) ranged from 10^1^ to 10^6^ molecules, while for DENV (r^2^ = 0.9816) the range was 10^2^ to 10^6^ molecules (Fig. 3). Four dilutions of positive controls were used as substrate to calculate the LOD with 95 % probability for each pathogen. The LOD for DENV was 35.30, for WNV 33.50, for *Rickettsia* 11.19, and for *Leptospira* 27.43 molecules.

The analytical specificity was evaluated by primer-BLAST. The analysis did not retrieve sequences of other unintended species. Furthermore, this analysis retrieved 1876 strains for DENV, 1000 for WNV, 302 for *Rickettsia* spp. and 479 for *Leptospira* spp. (Table 3). No cross-reactivity was detected in WNV, *Leptospira* or *Rickettsia* assays.

### Lyophilization assays

**Fig. 4:**
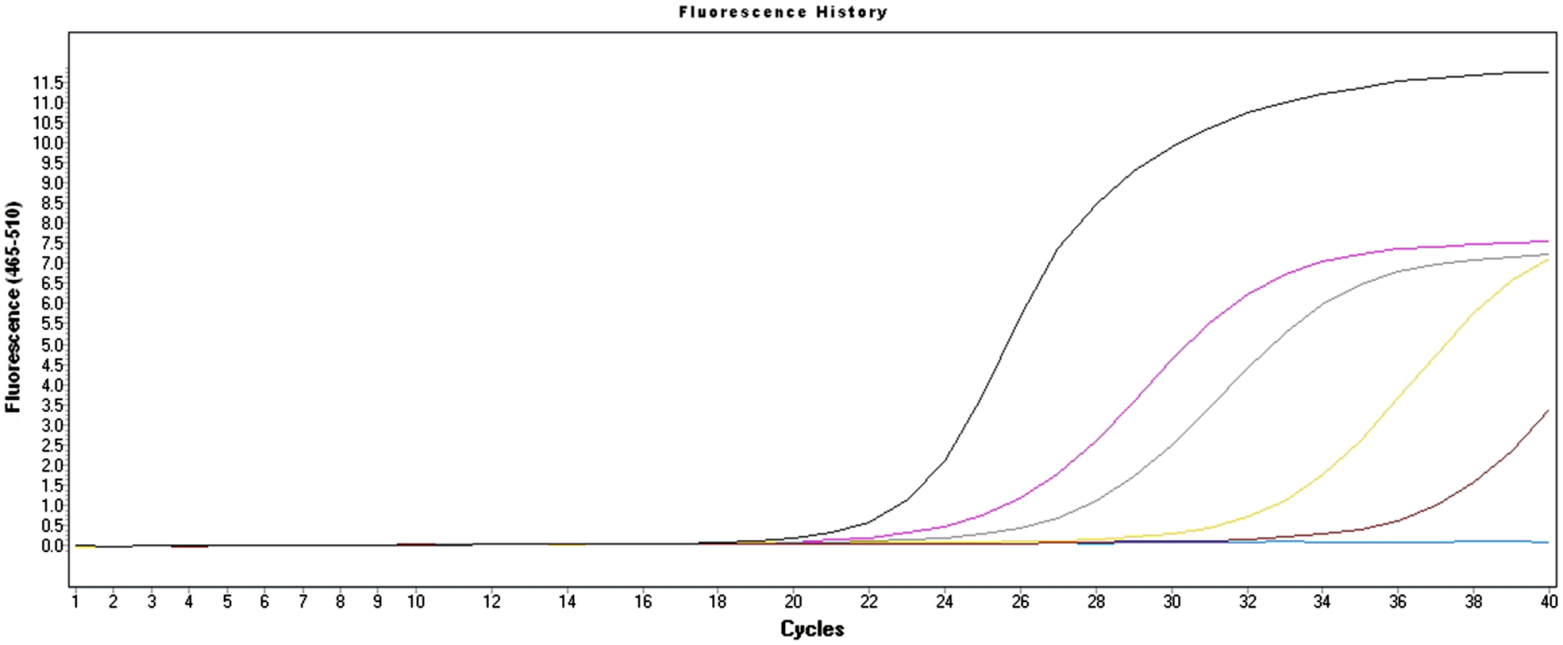
Amplification plots of the lyophilization assay. Fresh positive control showed a Cycle threshold (C_t_) of 22, trehalose 0 % C_t_ of 24, trehalose 2.5 % C_t_ of 26, trehalose 5 % C_t_ of 29 and trehalose 10 % C_t_ of 33

The fresh positive control reaction showed a C_t_ of 22 while the C_t_ of 0, 2.5, 5 and 10 % of trehalose treatments were 24, 26, 29 and 33, respectively (Fig. 4). All lyophilized treatments showed a drop in the maximum fluorescence, however, the lyophilized reaction without trehalose showed a smaller drop in fluorescence.

### Analysis of clinical samples

We performed a pre-validation of the diagnostic strategy presented herein, using 100 sera samples from patients with dengue-like symptoms collected during the last DENV outbreak (June-Nov 2015). From the 100 samples, 12 were positive for DENV and one sample was positive for leptospirosis. None of them were positive for rickettsiosis or WNV.

## Discussion

DENV, WNV, *Rickettsia* spp. and *Leptospira* spp. may coexist in warm and moist regions around the world, but often in Latin-American countries, only Dengue Fever has been recognized as a health problem. Because of this, we have developed a qPCR system that is intended for use in Latin American countries, where these four pathogens have been reported (e.g. Mexico). Since these pathogens produce similar symptoms and are circulating throughout the continent (Beeler et al. 2011; Elizondo-Quiroga et al. 2005; Gorrochotegui-Escalante et al. 2002; Ibarra-Juarez et al. 2012; Romer et al. 2011; Zavala-Velázquez et al. 1998), the actual number of infections might be currently misdiagnosed and treated only as Dengue Fever. Although cross reactivity between these viruses has been reported in immunological tests (Papin et al. 2004), and DENV and leptospirosis have been repeatedly confused (Brown et al. 2010; Ellis et al. 2008; LaRocque et al. 2005; Libraty et al. 2007; Papin et al. 2004), differential diagnostics is rarely conducted in these countries.

Individual DNA diagnostics have previously shown that nucleic acids of these four pathogens, can be found in serum samples only during the first 5 days of these diseases, and this also happens to be the most crucial time for diagnostic in patients, since severe complications or spontaneous resolution of the disease occur after the first week of the apparition of symptoms. After this time frame, antibodies can be found and serological tests can be used instead of molecular technics.

We have also developed a simultaneous qPCR-based system that uses novel primers for WNV, *Rickettsia* spp. and *Leptospira* spp., predicted to amplify most circulating strains of each agent and a pair of previously reported DENV primers. These four pairs of primers are able to amplify target sequences under the same PCR conditions. Additionally, since primers amplify conserved regions across strains of these four pathogens, the diagnostic result indicates the disease in the patient or sample, no matter the species or strain involved in the infection.

We employ cDNA controls for DENV and WNV assay, since assessing the analytical sensitivity using a constructed standard curve with viral titers would be prone to over-estimation given that viable and non-viable particles are detected. Researchers have also used cloned positive controls to determine the sensitivity of their tests (Hull et al. 2008; Kang et al. 2010), but this does not include the efficiency of reverse transcription and extraction process; therefore for diagnostic laboratories, we suggest to solve these drawbacks by using RNA from positive samples as controls.

In the analytical sensitivity estimations, we found a wide linear range of detection for our RT-qPCR assays and LODs of 35.30 copies for DENV, 33.50 copies for WNV, 11.19 copies for Rickettsia and 27.43 copies for Leptospira. Previous reports for DENV (Kim et al. 2015), for WNV (Linke et al. 2007), for Rickettsia (Renvoisé et al. 2012) and for Leptospira (Ferreira et al. 2014) have shown similar sensitivities. We have confirmed the selectivity using in silico tests, thus ruling out possible amplifications due to DNA of other microorganisms. Additionally, primers for DENV reported by Lai et al. showed no amplifications when tested against non-DENV RNA. Although our work has shown promising results so far to diagnose DENV and *Leptospira* spp., we have not been able to analyze this assay with positive samples from patients positive to *Rickettsia* spp. and WNV.

Dengue fever has emerged as a constant health threat to the population of Jalisco state, Mexico. According to official reports from Health Secretariat, 1229 cases of Dengue fever were reported during 2014 in this state, out of which 220 were from the metropolitan area. (‘Secretaria de Salud del Estado de Jalisco. Prevención Dengue’ 2015). In this work, out of 100 sera samples from patients with dengue-like symptoms, we found 12 positive to DENV in a period of 6 months from a single hospital. We additionally found one sample positive for leptospirosis. Interestingly, there were no official reports of any leptospirosis case for Jalisco state in 2015 (*Boletín Epidemiológico. Semana 52. Secretaría de Salud.*^2016^). In this study, we found no positive cases for WNV or rickettsiosis. From the total of samples, 87 were negative to the four diseases comprised in our diagnostic strategy; this could be due to inaccurate information provided by the patient to the health personnel of the hospital. Another possible explanation is the recent introduction of the Chikungunya virus into Mexico (Rivera-Avila 2014); since it is also an arbovirus that produces dengue-like symptoms and a very high attack rate has been reported (Renault et al. 2007) it is likely that some of the patients enrolled in this study, could have been infected with this virus.

Finally, we designed a prototype device consisting of a 96 well plate in which all necessary reagents for PCR, excluding nucleic acids, were added to each well and lyophilized. Every four wells of this device include the specific set of primers for DENV, WNV, *Rickettsia* spp*. and Leptospira* spp. According to this prototype, each sample must be added to a set of four wells in order to identify the nucleic acids in it. One plate can analyze up to 22 samples, one negative control and one positive control (Fig. 1).

Lyophilization of reagents into the plate provides several benefits: (a) it enhances the reproducibility of results, (b) it reduces the set-up time for reactions, (c) it reduces the risk of contamination and false positives, (d) it easies transport and storage of the plate. Apparently, components in the enzyme buffer are also a good cryo-protector and there is no need for additional enhancers to stabilize it during the processing of samples.

## Conclusions

At the moment the molecular differential diagnostic technic is necessary to clearly comprehend the epidemiological relevance of these diseases in some areas and may aid health secretariats and vector control departments to better aim their resources for effective management of outbreaks. Also, quick diagnosis will help to prescribe correct antibiotics for bacterial-origin fever, and a careful follow-up for fevers caused by DENV or WNV, therefore we are presenting a sensitive and selective kit that detects four relevant pathogens from tropical regions, that is also quick, cost-effective and easy to use.
